# Effect of gamified flipped classroom on improving nursing students’ skills competency and learning motivation: a randomized controlled trial

**DOI:** 10.1186/s12912-022-01096-6

**Published:** 2022-11-16

**Authors:** Mohamed E. H. Elzeky, Heba M. M. Elhabashy, Wafaa G. M. Ali, Shaimaa M. E. Allam

**Affiliations:** Medical Surgical Nursing Department, Faculty of Nursing, Mansoura University, Dakahlia, Egypt

**Keywords:** Gamification, Flipped classroom, Nursing students, Skills competency, Self-confidence, Learning motivation

## Abstract

**Background:**

Flipped learning excessively boosts the conceptual understanding of students through the reversed arrangement of pre-learning and in classroom learning events and challenges students to independently achieve learning objectives. Using a gamification method in flipped classrooms can help students stay motivated and achieve their goals.

**Methods:**

This study adopted a randomized controlled study design with a pre-test and post-test and involved 128 nursing students at Mansoura University. This study randomly divided the students into the study and control groups. Data were collected at three time points using six tools**.** In the intervention group, Moodle was gamified for 6 weeks.

**Results:**

A significant difference in the students’ self-confidence (*p* = 0.021), skills knowledge (*p* < 0.001), intensity of preparation (*p* < 0.001), and motivation (*p* < 0.001) was observed between the two groups; however, no difference in the students’ skills performance (*p* = 0.163) was observed between the two groups after using gamified flipped classrooms.

**Conclusions:**

Compared with the traditional flipped classrooms, gamified flipped classrooms improved nursing students’ motivation, intensity of preparation, skills knowledge, and self-confidence during laboratory clinical practice. Thus, gamification is a learning approach that can be implemented in conjunction with the flipped classroom model to motivate students to participate in the learning process.

Trial registration.

Prospectively registered with ClinicalTrials.gov on 26/04/2021; registration number NCT04859192.

**Supplementary Information:**

The online version contains supplementary material available at 10.1186/s12912-022-01096-6.

## Introduction

One of a nurse’s essential competences is the capacity to deliver care to patients based on nursing skills and knowledge [1]. Having satisfactory knowledge and performance of proper nursing skills improves students’ self-confidence while delivering care [2]. Nursing instructors have a crucial role in teaching students to masterly perform psychomotor skills [3, 4]. The flipped classroom (FC) strategy, is a highly learner-oriented pedagogical method [5] that allows educators to provide online materials for students to watch memorize and present independently before coming to classes so that the class time can be better spent on learning activities, like skills practice and discussions [6].

Many studies have confirmed that the FC strategy has a higher efficiency than traditional classrooms in enhancing nursing students’ skills, knowledge, attitude, having satisfactory study, self-learning, problem solving, and critical thinking [7–12]. Other studies emphasized that flipped learning improved nursing students' confidence in their ability to apply knowledge and skills in clinical practice [13–16]. However, other studies reported no significant differences in learning outcomes between the flipped and traditional classroom groups [17–19]. Many even felt that the FC strategy was less effective because more time was spent preparing outside class [20, 21].

To create a successful FC, students’ intrinsic motivation and compliance with the FC requirements are crucial components in achieving the preferred learning outcomes [10, 22, 23]. However, according to a literature review of studies on the FC strategy, the most faced difficulty is decreasing students’ motivation, which prevents the predicted increase in academic achievement [24, 25]. Moreover, a study by Heitz et al. [26] reported that 31% of students in the FC group were non-compliant. A survey regarding FC use before clinical skills laboratory teaching reported that the absence of student involvement with the FC strategy is one of the main challenges faced by educators [27]. According to a systematic review, the greatest obstacles for the instructors to overcome are how to engage and motivate students in watching the recorded lectures [28]. Moreover, another systematic review reported that the FC strategy may be perfectly applied to active learners, as opposed to passive learners, in terms of students’ learning satisfaction and skill performance [29]. Finally, a pre-class quiz fails to enforce students to watch pre-class videos regularly [30]. This suggests that to boost student participation during FC practice and prepare them for class, novel methods, such as gamification, are greatly needed [31].

The proper application of gamification strategies in student education is expected to serve as a tool for solving motivation and learning performance problems. First, the self-determination theory, which has been effectively applied to the setting of gamification, provides an explanation for the motivational appeal of various game design elements. Gamification uses game fundamentals, such as scores, leaderboards, and badges, toward non-game activities to increase student interest and motivation through competition [32–34]. Second, based on gamified learning theory; gamification can affect learning outcomes indirectly by improving already beneficial instructional content and influencing behaviors and attitudes [35].

The combination of FCs and gamification has been widely used in various studies, which have reported that this combination had a positive impact on student achievement. Sailer and Sailer [36] have shown that gamification of class activities improves social relatedness and motivation. Zhao et al. [37] have found a positive relation between gamification of electronic books and students’ FC performance, meta-cognition tendency, and motivation. Forndran and Zacharias [38] indicated that the students’ self-confidence had been positively impacted by gamified flipped learning. A systematic review on the use of gamified learning among university-level medical and nursing students reported that it positively influences student satisfaction and motivation [39].

Although FC learning is becoming increasingly popular, there are certain disadvantages that gamification can significantly address [40]. However, there are still very few studies on the gamification of FC in nursing education, and most have examined its effect on cognitive gains and psychological needs[31, 41, 42], and few have examined its effect on clinical learning outcomes of nursing students [43]. Axley [44] defined the principle of nursing competency from a wide angle. Essential attributes include motivation, attitude, critical thinking, maturity, openness, and self-evaluation, in addition to the sheer accomplishment of abilities. So, in this RCT study, we propose that gamification of FC (using game quiz, badges, leaderboards, levels, rank, and points) could motivate, increase preparedness before laboratory classes, and improve Fundamentals of Nursing students’ skills competency (knowledge, performance, and confidence).

## Aim of the study

This study was designed to assess the effects of using gamified FCs on the Fundamentals of Nursing students’ skills competency and learning motivation.

## Research hypothesis

(1) Nursing students learning in a gamified FC will have higher learning motivation scores than those learning in an FC only. (2) Nursing students learning in a gamified FC will be more prepared for fundamental skills laboratory than those learning in an FC only. (3) Nursing students learning in a gamified FC will have higher scores for fundamental skills knowledge than those learning in an FC only. (4) Nursing students learning in a gamified FC will have higher scores for fundamental skills performance than those learning in an FC only. (5) Nursing students learning in a gamified FC will have more confidence performing fundamental skills than those learning in an FC only.

## Conceptual framework

In the present study we based our hypothesis on two theories:

- I- According to Self-determination theory, people have three basic psychological needs that can motivate them to decide whether or not to engage in a particular activity: autonomy, relatedness, and competence [45]. Gamified activities that give individuals the autonomy to select the tasks they want to complete (e.g., by providing varying levels of difficult tasks; game quiz) can address this need.Participants' emotional and behavioral engagement can rise when they feel they have some degree of autonomy [46]. The need for people to interact or connect with one another is referred to as relatedness [45]. This demand is met by gamified activities that let participants compete or work together (Leaderboard and rank). An increased sense of relatedness aids in fostering feelings of enjoyment and can motivate individuals to continue taking part in the activity [46]. Competence is defined as the need to master one’s pursuits or learning. The use of immediate feedback (such as points or badges) and indicators of participants’ advancement (levels) can help boost individuals’ sense of competency [34].
- II- The gamified learning theory [35] provides a general framework that conceptualizes the relationship between gamification and learning. There are four elements to this theory: (1) instructional content (flipped videos), (2) behaviors and attitudes (intensity of preparation), (3) game characteristics (gamified quiz, badges, leaderboard, levels unlock, points) and (4) learning outcomes (skill competency). First, the theory suggests that, learners' behaviors and learning outcomes are directly influenced by the instructional content. Gamification is described as a method to enhance instruction rather than replacing it [35]. Second, the theory proposes that behaviors and attitudes affect learning outcomes. Third, game characteristics are expected to directly influence behaviors and attitudes [47].

## Methods

### Study design

A randomized controlled design with a pre-test and post-test and a control group was used in this study.

### Subjects

Participants were recruited from the Faculty of Nursing, Mansoura University. The students of the Fundamentals of Nursing II course (2020/2021) who were willing to participate were involved. This study required an a priori sample size of 128 students, who were randomly divided into two groups (study and control) using block randomization with a block size of 4. The sample size was determined using G*Power (version 3.1.9.7). Two-tailed t-test and two groups, with an effect size of 0.5, alpha of 0.05, and power of 80%, were previously identified. The effect size of 0.5 was obtained from a meta-analysis study which estimated a medium effect size in favor of gamification over learning without gamification [48]. Another study comparing motivation levels reported means of (20.72 and 19.72) among study and control groups respectively, with SD within each group equal to 2.95; using g power, it gives an effect size of 0.5 also [49]. Eligible students were selected using the inclusion criteria that included nursing students of both sexes who registered in the Fundamentals of Nursing II course in the second semester (2020–2021), who have access to the Internet at home, and who agreed to participate in the study**.** Those previously registered in the Fundamentals of Nursing II course were excluded. All randomization procedures were performed by an independent statistician and were blinded to the authors until intervention procedures.

### Tools

The primary outcomes were improvement in nursing students' motivation, skill knowledge, skill performance and skill confidence level. The secondary outcome includes improvement in nursing students' intensity of preparation. Six tools were used in this study.

#### Tool I: Questionnaire of demographic characteristics

This tool was used to obtain data regarding age, sex, economic status, grade point average, high school type, high school location, decision to join nursing, and level of interest in nursing. An 11-point scale (0 = lowest, 10 = highest) was used for student self-rating of economic status, degree of interest in the course, interest in the nursing profession, and class participation (Table 1).

**Table 1:** Frequency and percentage distribution of the students’ demographic characteristics (*n* = 64 in each group)

Variables	Study group *N* = 64		Control group *N* = 64		Significance
**Sex**	**No**	**%**	**No**	**%**	
Male	28	43.80%	33	51.6	*P* = 0.376
Female	36	56.20%	31	48.4	
**Secondary school place**					
Rural	42	65.6	36	56.2	*P* = 0.277
Urban	22	34.4	28	43.8	
**Economic status Mean** ± SD	6.5 ± 1.6		6.1 ± 1.6		*P* = 0.193^+^
**Range (0–10)**					
**Interest in Fundamental 2 course**	8.9 ± 1.3		8.7 ± 1.7		*P* = 0.312^+^
**Mean** ± SD					
**Range (0–10)**					
**Interest in nursing profession**	9.2 ± 1.2		8.8 ± 1.6		*P* = 0.113^+^
**Mean ± SD**					
**Range (0–10)**					
**Evaluation of degree of participation**	8.1 ± 1.6		7.8 ± 1.9		*P* = 0.394^+^
**Mean** ± SD					
**Range (0–10)**					
**Decision to join nursing**					
By yourself	49	76.6	44	68.8	*P* = 0.678
Advice from others	11	17.2	8	12.5	
**PLANS AFTER GRADUATION**					
Working as registered nurse	22	34.4	26	40.6	*P* = 0.568
Post graduate studies	31	48.4	25	39.1	
Change profession	0	0	1	0.02	
Other plans	11	17.2	12	18.8	

+ Independent t-test; chi-square test

#### Tool II: Instructional Materials Motivation Survey (IMMS)

The IMMS was developed by Keller [50]. This tool has four domains: relevance, attention, satisfaction, and confidence. The total number of questions was 36, which were answered using a five-point Likert scale (1, do not agree; 2, agree; 3, moderately agree; 4, agree; and 5, strongly agree). The expected scores from this questionnaire range from 36 to 180, with greater scores reflecting a greater level of motivation of learning.

#### Tool III: Confidence Scale (C-Scale)

The C-Scale was adopted from Grundy [51] and was used to estimate students’ confidence level relevant to skill performance. This tool consists of five items answered using a five-point Likert scale, and its total score ranges from 5 (low confidence) to 25 (high confidence).

#### Tool IV: Intensity of Preparation (IOP)

The IOP was adapted from Sailer and Sailer [36] and was used to assess preparation intensity using three items that cover the number of times students viewed the skills video, whether the users used the video lecture’s slides, and whether they took notes while watching the video lecture. The number of times the video lecture was viewed was counted by the students who responded to an open inquiry with their viewing history. The slides and note-related questions were scored as two dichotomous items, with the “yes” response receiving a value of 1 and the “no” response receiving a value of 0. To evaluate the variable preparation intensities, a pooled score was calculated for the aforementioned items.

#### Tool V: Fundamentals of nursing II knowledge tests

Three formative quizzes for each timepoint were developed by the researchers. Each consisted of 20 questions: quiz 1 covered oral medication administration and intramuscular injection; quiz 2 covered range of motion exercises and subcutaneous and intradermal injections. However, quiz 3 covered glucocheck and heat and cold applications. The scoring system was implemented by adding five points to every right answer and 0 points to every incorrect answer. Then, the total score was calculated.

#### Tool VI: Fundamentals of nursing II skill observation checklists

Eight checklists were developed after reviewing the literature, nursing textbooks, and logbook of the Faculty of Nursing, Mansoura University, which are as follows: checklist 1 (oral medication administration); checklist 2 (intramuscular injection); checklist 3 (subcutaneous injection); checklist 4 (intradermal injection); checklist 5 (range of motion exercises); checklist 6 (heat application); checklist 7 (cold application); and checklist 8 (glucocheck). The evaluation items were ranked using a three-point Likert scale (0 or omitted, totally incorrect; 1, partially correct performance; and 2, correct performance) [49].

### Validity and reliability

Tools II, III, and IV were translated into Arabic and back-translated to English. All tools were face validated by a jury of seven specialists in the medical surgical nursing field, and all tools had good validity with an average content validity index of 1.0. A pilot study was conducted involving 20 nursing students to test the clarity and reliability of the tools, and these students were excluded from the study. Cronbach’s α was calculated and was 0.93 for tool II, 0.84 for tool III, 0.89 for tool IV, and 0.95 for tool VI.

### Data collection

**Fig. 1:**
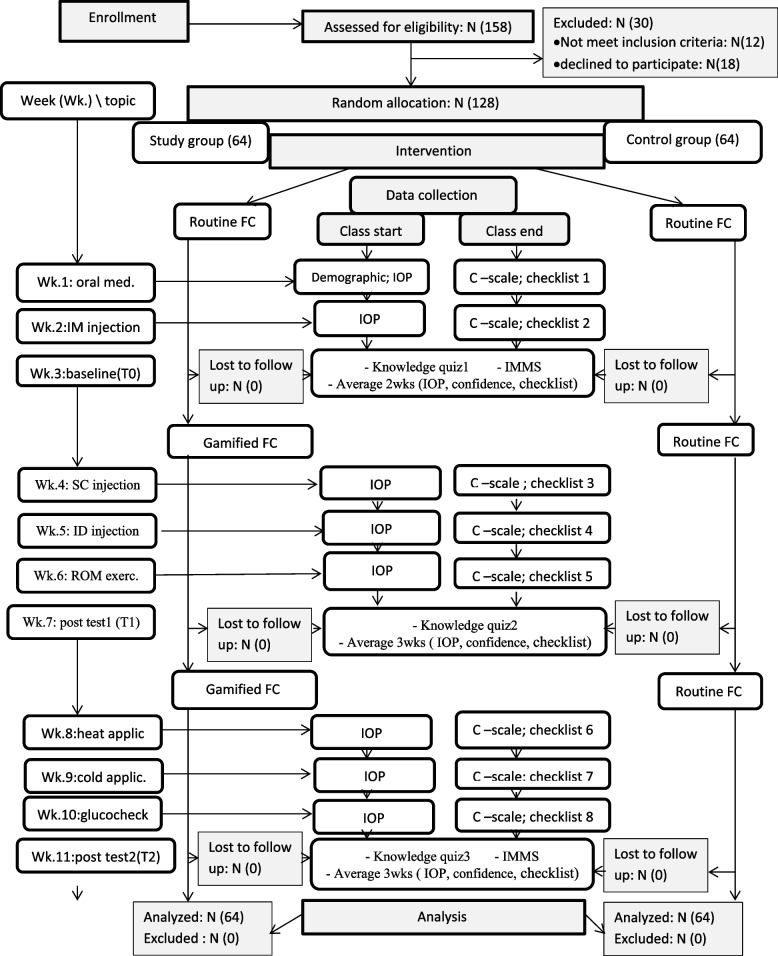
Schematic diagram representing the study protocol and data collection points

This was a single blinded trial where the data collectors were blinded to the study groups during the entire study period. After informed written consent was obtained, the students were randomly distributed to the intervention and control groups. The assistant staff gathered data for 11 weeks at three timepoints in the second academic semester (2020–2021) (Fig. 1). Timepoint 0 (T0) covers the first 2 weeks, and timepoints 1 (T1) and 2 (T2) cover the subsequent 3 weeks. Before each class session, tool IV was used to assess the IOP, and after each session, tools III and VI were used to assess the students’ confidence and skill levels. At the end of each timepoint, tool IV was used to assess the students’ knowledge level, and the mean overall IOP, confidence, and skill competency over weeks were calculated. Tool II was used to assess the students’ motivation at T0 and the end of the study. Data collectors were trained on assessment tools and ensure no missed data during evaluation.

### Intervention

**Fig. 2:**
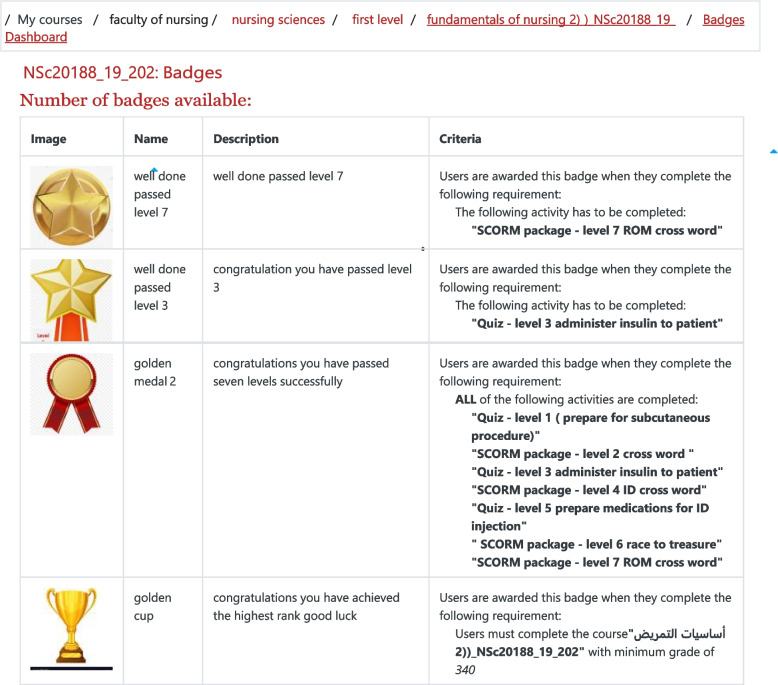
Snap shot of fundamental of nursing badges

The two groups were taught once a week (120 min) in the laboratory skills with the same six instructors and content. In the first 2 weeks, both groups received routine FC instructions, which included a 2-h skill video and one multiple choice quiz related to pre-class materials, which were uploaded to the Moodle page 1 week before clinical lab training, whereas class activities included demonstration by the instructor and re-demonstration of skills on simulators by the students; 3 case scenarios; and a peer evaluation checklist. Then, after collecting baseline data, Moodle was gamified over the remaining 6 weeks for the students in the intervention group, and game elements included three gamified quizzes on each skill (i.e., easy, moderate, and high difficulty), badges, leaderboards, ranks, levels/unlocks, and points (Fig. 2). The gamified activities were shown to the intervention group only, and the students in the intervention group were instructed to not share their accounts with other students until the completion of this study. The students could compete and make maximum points and badges through this online motivation. The instructor used the Active Presenter and Hot Potatoes software and game module in designing the gamified quizzes. The quiz contained videos and images along with the text. Several question classifications (e.g., matching, true/false; multiple choices, drag and drop, and fill in the blanks) and several quiz forms (e.g., crosswords, race to treasure, and millionaire quiz) with a total of 18 game quizzes and levels were used in the course. For the study participants, 25 points were given for watching the course materials, and 5 points were given for each correctly answered question in gamified quizzes. Easy game quizzes contain 5 questions; a moderate quiz consists of 10 questions in crossword form; and a difficult quiz contains 15 questions in millionaire quiz form. Students who passed a difficult quiz were given an extra 25 points. The system would automatically provide the pupils with a new online badge after they reached 100 points. Each level unlocks after passing the previous level. Students with the highest rank in each level were provided with another medal badge, and for each set of levels, students were awarded with a new badge as well. Therefore, the more the students took the game quizzes and passed them, the more points they earned and the more badges they got. However, the control group and students who didn’t agree to participate in the program did not receive any intervention, except for their routine FC education. The game activities were also shown to the control group at the end of the study, after the post-test phase, and before final exams. The dosage of gamification in our study was based on Sanchez, Langer, and Kaur (2020) [52], who used several game quizzes on each topic, for a total of 34 game quizzes over 13 weeks. Furthermore, the quiz difficulty was based on (Aşksoy, 2018) [24], who used different quiz difficulties for each topic. The number of questions per game quiz in the literature varies between 5 (Sanchez, Langer, and Kaur (2020) [52] and 20 (Zainuddin, 2018) [31].

### Statistical analysis

Descriptive statistics (i.e., mean, standard deviation, and frequency) and inferential statistics (i.e., paired t-test, independent t-test, repeated-measures analysis of variance [ANOVA], and chi-square test) were used. Moreover, four 3 × 2 repeated-measures ANOVAs were performed. The dependent variables were skills performance, confidence, knowledge, and IOP; the independent variables were time (T0, T1, and T2) and group (intervention or control). The outcomes of the program were evaluated by comparing the rating scale results according to (a) the test period (T0 vs. T1 and T1 vs. T2) using a contrast test with the pre-test as the reference and (b) the group (study vs. control) at the three test periods. Data analysis was performed using Statistical Package for the Social Sciences, version 20, and the significance level was set at *p* < 0.05. There were no missing data. For this study, using 2 way repeated measures ANOVA and t test was appropriate without fear of violating assumptions of normality because the central limit theorem applied when samples in both groups were > 30 [53–55].

## Results

### Demographic characteristics

Data on the demographic characteristics of the groups were checked for homogeneity (Table 1). No substantial differences in sex, secondary school location, economic status, interest in the Fundamentals of Nursing course, interest in the nursing profession, degree of participation, Fundamentals of Nursing I grade, decision to join the nursing profession, and plans after graduation were observed between the two groups.

### Skills performance, confidence, knowledge, and IOP scores

Table 2 presents the means and standard deviations of the four dependent variables under study at the three test periods along with the repeated-measures ANOVA results for the dependent variables.

**Table 2:** Repeated-measures analysis of variance of skills performance, confidence, knowledge, and intensity of preparation among both groups throughout the study (*n* = 64 in each group)

**Variables**	**Time**	**Means** ± **SD**		**F**	**df**	P^*^	**η**_**p**_^**2**^
		Study *N* = 64	Control *N* = 64				
**Skills performance**	**T0**	81.2 ± 7.9	79.9 ± 8.8	0.929	2.252	0.396	0.007
	**T1**	82.1 ± 8.2	79.8 ± 9.4	1.972	1.126	0.163	0.015
	**T2**	82.5 ± 7.5	80.1 ± 10.3	0.614	2.252	0.542	0.005
**Skills Confidence**	**T0**	19.7 ± 2.9	19.2 ± 3	12.9	1.817	< 0.001^B,C^	0.093
	**T1**	20.6 ± 2.2	19.5 ± 3	5.496	1	0.021^B^	0.042
	**T2**	21.1 ± 2	19.7 ± 3.1	3.418	1.817	0.039^C^	0.026
**Skills Knowledge**	**T0**	80.9 ± 9.7	79.9 ± 7.5	24.436	1.713	< 0.001^A,B^	0.162
	**T1**	86.1 ± 8.8	80.4 ± 6.7	17.264	1	< 0.001^A,C^	0.121
	**T2**	89.1 ± 7.1	81.2 ± 7.9	12.962	1.713	< 0.001^B,C^	0.093
**Intensity of preparation**	**T0**	2 ± 1.2	1.8 ± 1	30.97	2	< 0.001^A,B^	0.197
	**T1**	2.8 ± 1.2	1.9 ± 0.9	33.43	1	< 0.001^A,C^	0.21
	**T2**	3.6 ± 1.4	2 ± 1.2	18.04	2	< 0.001^B,C^	0.125

*SD* standard deviation, *T0* pre-test, time effect, *T1* 1st post-test, group effect, *T2* 2nd post-test, time*group effect, * one-tail significance test

^A, B,C^significant differences between the corresponding groups by Bonferroni post hoc multiple comparisons

Regarding skills performance, repeated-measures ANOVA revealed an insignificant difference in time (*F*(2.252) = 0.929, *p* = 0.396, **η**_**p**_^**2**^ = 0.007), an insignificant time*group effect (*F*(2.25) = 0.614, *p* = 0.542, **η**_**p**_^**2**^ = 0.005), and an insignificant difference between the two groups (*F*(1.12) = 1.972, *p* = 0.163, **η**_**p**_^**2**^ = 0.015).

Regarding skills confidence, repeated-measures ANOVA revealed a significant difference in time (*F*(1.81) = 12.9, *p* < 0.001, **η**_**p**_^**2**^ = 0.093), a significant time–group effect (*F*(1.81) = 3.418, *p* = 0.039, **η**_**p**_^**2**^ = 0.026), and a significant difference between the two groups (*F*(1) = 5.496, *p* 0.021, **η**_**p**_^**2**^ = 0.042). Post hoc analysis with Bonferroni adjustment revealed that the students’ confidence level increased significantly from T0 to T1 and from T0 to T2, but not from T1 to T2.

Regarding skills knowledge, repeated-measures ANOVA revealed a significant difference in time (*F* (1.71) = 24.4, *p* < 0.001, **η**_**p**_^**2**^ = 0.162), a significant time–group effect (*F* (1.71) = 12.962, *p* < 0.001, **η**_**p**_^**2**^ = 0.093), and a significant difference between the two groups (*F* (1) = 17.264, *p* < 0.001, **η**_**p**_^**2**^ = 0.121). Post hoc analysis with Bonferroni adjustment revealed that the students’ knowledge level increased significantly from T0 to T1, from T0 to T2, and from T1 to T2.

Regarding skills preparation intensity, repeated-measures ANOVA revealed a significant difference in time (*F* (2) = 30.97, *p* < 0.001, **η**_**p**_^**2**^ = 0.197), a significant time–group effect (*F*(2) = 18.04, *p* < 0.001, **η**_**p**_^**2**^ = 0.125), and a significant difference between the two groups (*F*(1) = 33.34, *p* < 0.001, **η**_**p**_^**2**^ = 0.210). Post hoc analysis with Bonferroni adjustment revealed that the students’ IOP increased significantly from T0 to T1, from T0 to T2, and from T1 to T2.

### Motivation scores

No statistically significant difference in the mean pre-test motivation score was observed between the two groups (Table 3). However, the mean post-test motivation score was significantly higher in the intervention group than in the control group (*p* < 0.001). Moreover, a statistically significant difference was observed between the mean pre-test and post-test motivation scores in the intervention group (*p* < 0.013); however, no significant difference was observed between the mean pre-test and post-test motivation scores in the control group.

**Table 3:** Comparing pre- and post-intervention motivation scores between the study and control groups: (*n* = 64 in each group)

	Study mean ± SD		Control mean ± SD		P1	P2	P3	P4
**MOTIVATION domains**	**T0**	**T2**	**T0**	**T2**				
Attention	44.4 ± 11.1	48.9 ± 10	42.2 ± 11.6	41.2 ± 9.9	0.279	< 0.001	0.013	0.400
Relevance	36.3 ± 5.9	38.5 ± 7.1	34.4 ± 5.8	33.8 ± 5.4	0.072	< 0.001	0.028	0.366
Confidence	33.4 ± 5.8	35.3 ± 7	31.7 ± 5.7	31.5 ± 5.3	0.087	0.001	0.044	0.739
Satisfaction	23 ± 5	24.7 ± 4.4	21.8 ± 4.7	21.3 ± 3.8	0.162	< 0.001	0.011	0.208
Total learning motivation score	137.1 ± 23.8	147.1 ± 24.1	130.1 ± 24.2	127.7 ± 19.8	0.102	< 0.001	0.013	0.394

P1 t baseline study and control

P2 t post study and control

P3 paired t test pre and post for study group

P4 paired t test pre and post for control group

### Correlation among the measured variables after gamified FC intervention

At T2, a significant positive correlation was found between learning motivation and skills knowledge (*r* = 0.201, *p* < 0.022). Additionally, a significant positive correlation was observed between confidence and skills knowledge (*r* = 0.373, *p* < 0.002) and skills performance (*r* = 0.247, *p* < 0.049). Moreover, a significant positive correlation was observed between skill performance and skills knowledge (*r* = 0.409, *p* < 0.001). Lastly, a significant positive correlation was observed between IOP and skills confidence (*r* = 0.306, *p* < 0.014), skills knowledge (*r* = 0.368, *p* < 0.003), and skill performance (*r* = 0.359, *p* < 0.004) (Table 4).

**Table 4:** Pearson Correlation Coefficients among the measured variables after gamified intervention at T1 and T 2 points in the study group

Variables	T 1				T 2			
	**1**	**2**	**3**	**4**	**1**	**2**	**3**	**4**
	**r (p)**	**r (p)**	**R (p)**	**r (p)**	**r (p)**	**r (p)**	**r (p)**	**r (p)**
1.learning motivation								
2.confidence	0.092 (.471)				0.201 (.112)			
3.skills knowledge	0.181 (.152)	0.296 (.018)^*^			0.201 (.022)^*^	0.373 (.002)^*^		
4.skills performance	0.188 (.137)	0.065 (.608)	0.290 (.020)^*^		0.116 (.363)	0.247 (.049)	0.409 (.001)^*^	
5.IOP	0.231 (.066)	0.294 (.018)^*^	0.256 (.041)^*^	0.44 (< .001)^*^	0.206 (.103)	0.306 (.014)^*^	0.368 (.003)^*^	0.359 (.004)^*^

Significant < 0.05

## Discussion

The nursing education focus is on enhancing learners’ intrinsic motivation and improving their skills competency in the field of nursing. In this study, it is expected that the participants in the intervention group will have better skills performance. However, although the intervention group had higher scores than the control group, the difference was not statistically significant. This result agrees with the results of Mekler et al. [33] and Sailer and Sailer [36], who reported that the elements of the games (i.e., game quiz, badges, and leaderboard) did not significantly affect the competence of the students. However, this is in contrast with the findings of Lai et al. [56], who reported a significant improvement in practical skill scores in medical students of the study group, and this may be because group teaching was face to face (lectures and hands on training). Furthermore, gamification activities were administered in class, and the researcher used gamification elements, in addition to game-based learning strategies (3 games) during the class. Another study by Kim and Kim [43] reported that gamified FC learning increases students’ empathy with patients; this may be because gamification was used in and out of classes, and the researcher combined gamification with other teaching methods, including situation-based learning; however, this study compared gamified learning with traditional learning, and the study groups were from different academic years. This may raise the questions of whether gamifying laboratory skill training sessions (in and out of class) and whether adding serious games in addition to gamification to FC learning would help improve skill scores compared with FC. Furthermore, in our study, the students could view the skill videos and practice the gamified activities 1 week before each procedure, and their levels of skill performance were assessed immediately. Students at the fundamental level need many training and practice time. Hence, to differentiate skills between the two groups, 1 week may not be adequate [49].

In this study, a positive correlation was found between skill performance, skills knowledge, and confidence. This result agrees with those of Tan et al. [7], who also reported a significant positive correlation between knowledge, skill performance, and confidence. In contrast, a study by Lee et al. [49] reported an insignificant correlation between knowledge, skill performance, and confidence. This may be because in this study, mobile-based video learning did not significantly affect the students’ knowledge or skill performance; moreover, most study participants were females (approximately 90%), whereas, in our study, approximately half of students were males. The self-efficacy of learners in using digital devices has been linked to sex differences as a potential influencer [57]. Furthermore, motivation behind gamification engagement is different between sexes, and males play more games; thus, competition is a great component for male students [58]. This raises the question: which game elements are appropriate for students based on sex differences?

In terms of confidence, a significant difference was found between both groups, and this was supported by Forndran and Zacharias[38] and Ekici [40], who reported that the students’ self-confidence was positively impacted by gamified FC learning. This finding also agrees with those of Sung and Hwang [59], who reported that students of gamified classes felt more confident, competent, and engaged in classroom activities. In our opinion, this is because the students were given instant task-level feedback, which have been proven to help boost students’ confidence during the gamified intervention [31].

The results of this study indicated that gamification has a strong positive effect on the students’ skills knowledge. As reported by Zainuddin [31], the gamification of quizzes made pre-class content easier for students to be seriously learned. A study by Lai et al. [56] involving medical students reported significant improvements in the knowledge of the students in the gamified group. Also, our findings are consistent with those of [60–63] who reported a significant improvement in the knowledge level of the gamified group. In contrast, a study by Trevino et al. [64] and Lee et al. [65] reported that educational games had a similar impact on the knowledge level as attending an engaging, didactic lecture. Moreover, a study by Selby et al. [66] reported that the interactive lecture group had better knowledge than the game group, and this was interpreted as during game playing, the students have other distractions, whereas lectures forced students to focus on facts. However, those three studies used game-based learning, not gamified FC learning.

Another important implication emerging from these study findings is the IOP, which was significantly different between both groups. Furthermore, our results demonstrated significant correlations among the students’ IOP, skill knowledge, skill performance, and skill confidence. This finding agrees with those of Poondej and Lerdpornkulrat [67], Mohamed and Lamia [68] and Huang et al. [41] who proved that students in a gamified FC environment were more likely to complete homework and other pre- and post-class assignments on time than those in a non-gamified environment. Additionally, Jo et al. [69] applied gamification to an FC and were successful in raising students’ preparation. Another study by Sailer & and Sailer [36] reported that the students’ preparation level had a great impact on the performance of the learning process. Therefore, gamification can be an intervention to support extracurricular activities and thereby promote student preparation [69].

After gamification, the study group’s motivation levels in all four areas (i.e., relevance, attention, satisfaction, and confidence) were significantly higher than those of the control group. This finding agrees with that of Inangil et al. [60], who reported significantly higher attention, satisfaction, and total motivation score among the nursing students in the gamified group than control group. Other studies by Aşıksoy [24] and White and Shellenbarger [70] reported that gamified FCs increase student motivation and that badges guaranteed that students actively participated in classroom activities, made competitive environments in a positive way, enhanced peer relationships and generate formative feedback. Another study by Sailer and Sailer [36] reported that a gamified quiz can increase students’ feelings of social belonging and intrinsic motivation. Matching with this result, many studies have reported that education gamification positively affects participation and motivation [39, 42, 60, 71–73]. In contrast, a study by Mekler et al. [33] reported that motivation was not affected by gamification. Motivational shortage due to the implementation of gamification was because the required tasks to complete could hardly be considered challenging and game elements (i.e., points, leaderboards, and levels) were evaluated separately for three groups of students. These findings agree with those of Sailer and Sailer [36], who reported that the choice of questions and the gamified quiz design are critical because motivation might differ depending on the quiz difficulty.

## Limitations

However, this study has many limitations. First, there are few similar randomized controlled trials with which to compare our results. Most randomized controlled trials were not comparable because they either used game-based learning or the control group used conventional teaching and not FC learning and the subjects were not usually higher education nursing students. Furthermore, it is noted that the terms game-based learning and gamification are used synonymously in many studies, despite their mechanism differences. Second, although the pilot reliability was assessed in the preliminary test, variance among the three evaluators was inevitable and might affect the results. Moreover, the results cannot be generalized on a national scale because the students under study were from a single university. Furthermore, the Hawthorne effect cannot be ignored because the participants were aware that they were under assessment to evaluate the effectiveness of gamification, which thus may have led to a bias. Finally, passive learners may attempt to ask active students about the answers and achieve badges also without watching the video lessons and this further affect the skill scores. Although game quizzes add fun to the course and foster collaboration and competition among the students, other forms of gamification challenges may be required to obtain valid results of the students’ learning achievements in a gamified FC course.

## Conclusion and recommendations

This study demonstrated that Fundamentals of Nursing students learning in a gamified FC had better skills knowledge, confidence, and motivation and were well prepared before clinical classes than those learning in a conventional FC. The gamification elements in this study (i.e., badges, game quizzes, leaderboards, points, level unlock, and ranks) created a positive competitive environment and fostered the students’ motivation. Gamified FC learning can be considered an effective teaching method for delivering learning materials to nursing students to enhance their motivation and skills competence.

However, further research must compare gamified FCs with FC learning; examine its effect on learning outcomes, student satisfaction, and confidence; and explore factors affecting gamified FC learning outcomes in nursing education. Future studies are necessary to ascertain whether sex differences or individuals in some subgroups, such as students having low academic degrees, may earn more from participating in gamified FC learning. Future studies should compare both gamified FC and game-based FC interventions with each other and with controls and evaluate psychomotor skill acquisition and patient outcomes. Moreover, they should investigate the combining effects of gamification and serious games in FC learning and their effects on skill scores. Furthermore, these studies should compare in and out of class gamification in FC learning or the combination of both and their effect on learning outcomes. To increase the validity of the effectiveness of the gamification technique compared with the traditional method, a more thorough assessment process, including final test scores, should be performed. The long-term effects of gamification are important to be studied, to evaluate the long-term effects of game designs on user behavior.

## Supplementary Information

**Additional file 1.** Database.

## Data Availability

“The dataset supporting the conclusions of this article is included within the article (and in the Additional file 1)”.

## Abbreviations

FC: Flipped classroom
IMMS: Instructional Materials Motivation Survey
C- Scale: Confidence Scale
IOP: Intensity of Preparation
